# Artificial Intelligence and Hand Hygiene Accuracy: A New Era in Infection Control for Dental Practices

**DOI:** 10.1002/cre2.70150

**Published:** 2025-05-26

**Authors:** Salwa A. Aldahlawi, Amr H. Almoallim, Ibtesam K. Afifi

**Affiliations:** ^1^ Department of Basic and Clinical Oral Sciences, Faculty of Dental Medicine Umm Al ‐ Qura University Makkah Saudi Arabia; ^2^ Independent Scholar Riyadh Saudi Arabia; ^3^ Department of Medical Microbiology and Immunology, Faculty of Medicine Tanta University Egypt

**Keywords:** artificial intelligence, convolutional neural network, dental clinics, hand hygiene, infection control, machine learning

## Abstract

**Objective:**

The study aimed to assess the efficacy of an artificial intelligence (AI) model in evaluating hand hygiene (HH) performance compared to infection control auditors in dental clinics.

**Material and Method:**

The AI model utilized a pretrained convolutional neural network (CNN) and was fine‐tuned on a custom data set of videos showing dental students performing alcohol‐based hand rub (ABHR) procedures. A total of 66 videos were recorded, with 33 used for training and 11 for validating the model. The remaining 22 videos were designated for testing and the AI‐ infection control auditors comparison experiment. Two infection control auditors assessed the HH performance videos using a standardized checklist. The model's performance was evaluated through precision, recall, and F1 score across various classes. The level of agreement between the auditors and the AI assessments was measured using Cohen's kappa, and the sensitivity and specificity of the AI were compared to those of the infection control auditors.

**Results:**

The AI model has learned to differentiate between classes of hand movement, with an overall F1 score of 0.85. Results showed a 90.91% agreement rate between the AI model and infection control auditors in evaluating HH steps, with a sensitivity of 85.7% and specificity of 100% in identifying acceptable HH practices. Step 3 (back of fingers to opposing palm with fingers interlocked) was consistently identified as the most frequently missed step by both the AI model and the infection control auditors.

**Conclusion:**

The AI model assessment of HH performance closely matched auditors' evaluations, suggesting its reliability as a tool for evaluating and mentoring HH in dental clinics. Future research should explore the application of AI technology in different dental settings to further validate its feasibility and adaptability.

## 1 Introduction

Hand hygiene (HH) is among the most critical pillars of patient safety and healthcare quality assurance (World Health Organization 2009). It is strongly recommended by the World Health Organization (WHO) as a key performance indicator and a fundamental requirement for infection control programs in healthcare settings. The COVID‐19 pandemic has further highlighted the critical role of effective HH practices in reducing transmission risks and their significance in implementing preventive measures (World Health Organization 2021; Hansen 2022). Alcohol‐based hand rub (ABHR) is considered the most efficient preventive method for reducing healthcare‐associated infections (Lotfinejad et al. 2021).

WHO guidelines identify five essential moments of HH and recommend six practical steps for proper HH (World Health Organization 2009). However, previous studies on HH audits have predominantly focused on monitoring compliance with the five moments rather than evaluating the quality of the six recommended steps (Szilágyi et al. 2013). Low‐quality HH, even with high compliance, can increase the risk of infection (Cheng et al. 2019). To assess HH performance quality, past approaches have included direct observation (Guanche Garcell et al. 2017), monitoring consumption of products (World Health Organization 2009), and bacterial counts from fingertip prints (Lingawi et al. 2017). However, microbiological testing is time consuming and susceptible to cross‐contamination from the environment, potentially compromising the results (Szilágyi et al. 2013; Wang et al. 2021). Similarly, tracking soap or hand sanitizer consumption may not reliably reflect actual usage due to factors like product efficacy, environmental conditions, and the potential for overuse or waste (Bittner et al. 2002).

While direct observation allows for real‐time interaction between practitioners and auditors, enabling immediate correction of improper practices, it can also create tension. This awareness of being observed, known as the Hawthorne effect, may lead to changes in behavior (Purssell et al. 2020). Additional challenges of direct observation include inter‐rater reliability and validity, concerns over patient privacy, and disruptions to workflow due to the proximity of auditors to handwashing stations (Schaffzin 2022). Furthermore, this method can be time‐consuming and may only allow for evaluating a small sample of practitioners (Deochand and Deochand 2016).

Given the limitations of traditional methods and the increasing pressure from accreditation bodies to measure and document compliance and the quality of HH as part of healthcare quality assurance, there is an urgent need for alternative assessment methods (Hansen 2022).

One potential approach is using camera systems or 3D gesture trackers to monitor fine hand movements and identify user gestures. These systems can provide feedback to users or a central management system, aiming to create an automated tool to ensure compliance with HH guidelines (Singh et al. 2020). However, despite the high accuracy rates reported in the literature, transitioning these technologies to real‐world applications for HH quality monitoring has proven to be challenging (Lulla et al. 2021; Elsts 2022; Zhang et al. 2023).

Artificial intelligence (AI) is emerging as a transformative force across multiple sectors, including medicine and dentistry, with potential applications in infection control and prevention (Fitzpatrick et al. 2020). In dentistry, AI is used for diagnosis, clinical decision‐making, treatment planning, and prognosis prediction (Ghods et al. 2023). In the context of infection control, AI holds promise for diagnosing infections, predicting high‐risk patients during epidemics, and enhancing HH practices. AI‐driven tools with high‐quality data sets for HH education and auditing could significantly improve compliance and foster behavior change in healthcare settings (Fitzpatrick et al. 2020).

Therefore, the aim of this study was to investigate the effectiveness of an AI model in detecting adequate HH performance compared to infection control auditors' evaluations of HH quality among dental students at the dental teaching hospital, Umm Al‐Qura University.

## 2 Materials and Methods

**Figure 1:**
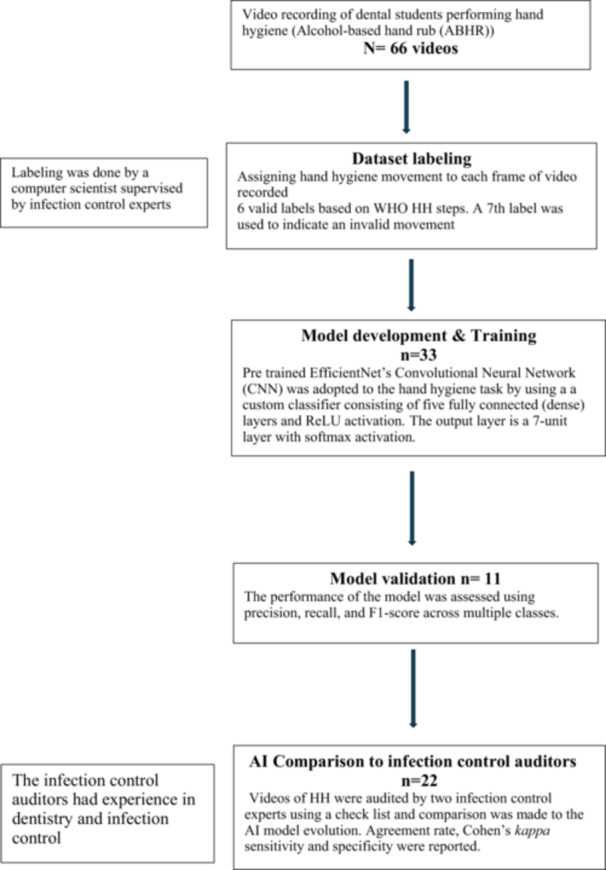
The workflow of the study.

The study was carried out at the dental clinics of the dental teaching hospital, Umm Al‐Qura University (UQUDENT). All participants were asked to provide a signed written consent before participating in the study. Ethical approval was obtained from the Institutional Review Board of Umm Al‐Qura University (No. HAPO‐02‐K‐012‐2024‐02‐2046). A summary of the method is presented in Figure 1.

### 2.1 UQUDENT Data Set

The HH movement data set was created by video recording dental students and dental assistants performing ABHR procedure.

The video recording device used was a wall‐mounted GoPro Hero12 camera set to 120fps and 460 × 460 resolution. To maintain the anonymity of the participants, the camera was mounted in such a way that only the hand movements were recorded. All recordings took place in one dental clinic.

Subjects were instructed to put their hands in clear sight of the camera and begin the ABHR procedure. The camera was controlled through a mobile application that allowed the start and end recording for each participant.

The videos were subsequently labeled and formed as the data set used to train and test our AI model (Table 1).

**Table 1:** Composition of data set by classes. Six classes were identified corresponded to steps 0–5 in HH procedure by WHO. An unknown class was added for unidentified hand movement.

Class	Footage amount (s)	Total action segments	Number of videos appeared
0	128.2	87	52
1	152.2	103	44
2	98.1	62	41
3	74.7	38	20
4	122.1	95	41
5	101.1	78	37
6 = Other/unknown	730.5	414	55
**Total**	**1406.9**	**877**	

### 2.2 Model Development

**Figure 2:**
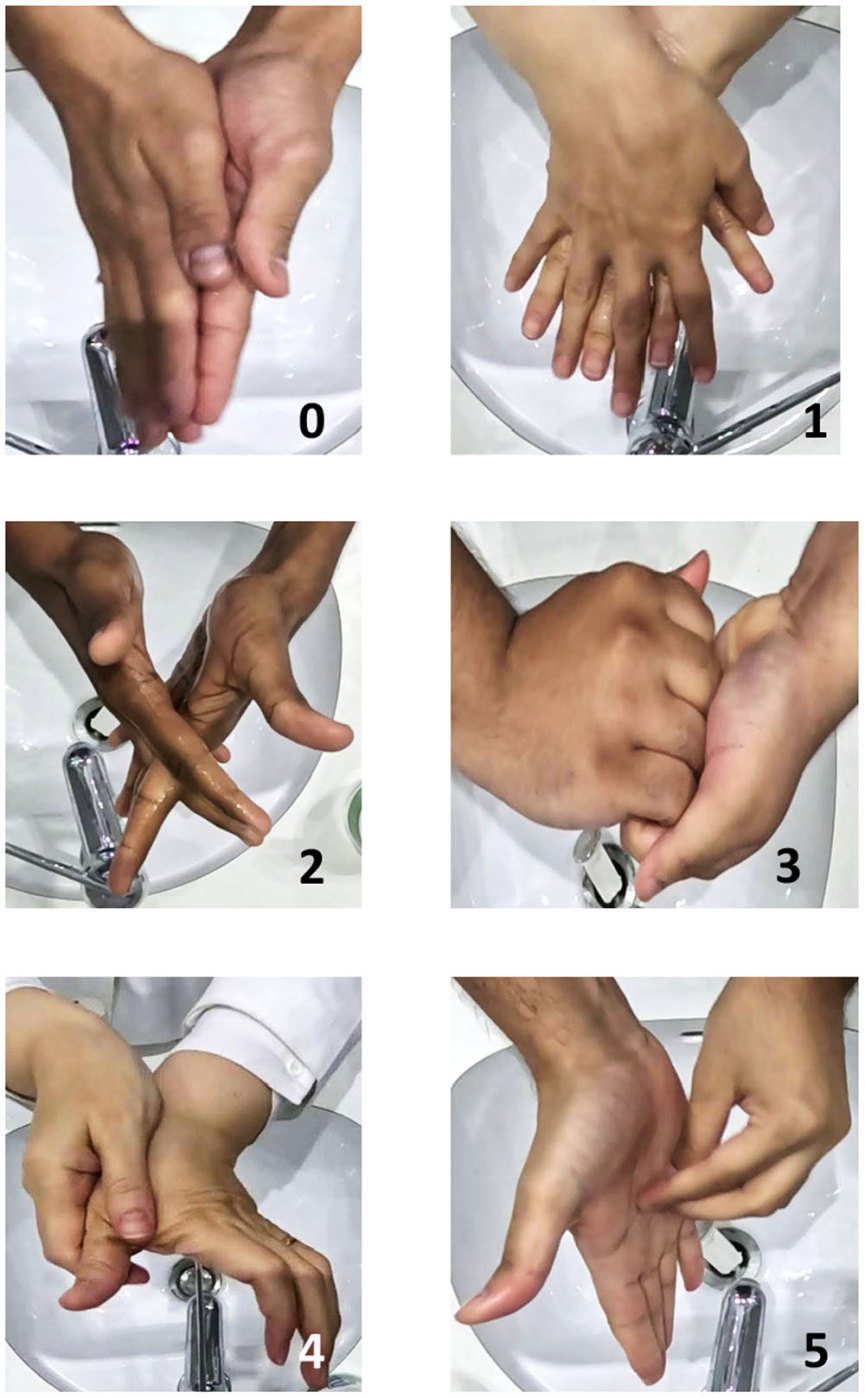
Hand hygiene steps. Step 0: Palm to palm. Step 1: palm over dorsum with interlaced fingers. Step 2: Palm to palm with figure interlaced. Step 3: Back of fingers to opposing palm with fingers interlocked. Step 4: Rotation rubbing of thumb. Step 5: Rotational rubbing with tip of fingers.

First, the videos were labeled. The labeling procedure consisted of assigning an HH movement to each frame of the video recorded. In total, six valid labels were created based on the guidelines provided by the WHO Handrub Procedure (Figure 2). A seventh label was used to indicate an invalid movement. The labeling was conducted by a computer scientist supervised by an infection control expert.

We utilized EfficientNet's convolutional neural network (CNN) as the base of our AI model (Fitzpatrick et al. 2020), leveraging its pre‐trained weights from ImageNet. To adapt the pre‐trained EfficientNet model to the HH task, we append a custom classifier consisting of five fully connected (dense) layers and ReLU activation (Ghods et al. 2023). Each dense layer is followed by a dropout layer to prevent overfitting. The output layer is a 7‐unit layer with softmax activation. The model summary is presented in Table 2.

**Table 2:** Model architecture summary.

Layer Type	Shape	Params
InputLayer	[(None, 240, 240, 3)]	0
Sequential	(None, 120, 120, 3)	0
Sequential	(None, 120, 120, 3)	0
Efficientnetv2‐s	(None, 4, 4, 1280)	20331360
GlobalAveragePooling2D	(None, 1280)	0
Dense	(None, 128)	163968
Dropout	(None, 128)	0
Dense	(None, 128)	16512
Dropout	(None, 128)	0
Dense	(None, 128)	16512
Dropout	(None, 128)	0
Dense	(None, 128)	16512
Dropout	(None, 128)	0
Dense	(None, 128)	16512
Dropout	(None, 128)	0
Dense	(None, 7)	903

### 2.3 Preprocessing

The videos were partitioned into training, validation, and test subsets following a 60%, 20%, and 20% split, respectively. Each video was decomposed into individual frames. To reduce training time and mitigate redundancy, we implemented a frame sampling strategy where we selected one frame out of every ten consecutive frames. For compatibility with EfficientNet, the images were resized to 120 × 120 pixels, and pixel values were normalized to the range [0, 1]. Data augmentation techniques, including rotation, scaling, and flipping, were applied to enhance the diversity of the training data and mitigate the risk of overfitting. To handle the imbalance of classes in the data set, we calculated class weights, which were passed into the Keras framework during model training.

### 2.4 Training Process

The training process was executed in two phases. First, we employed transfer learning by freezing the EfficientNet layers and training only the appended dense layers. This strategy allowed the model to acquire task‐specific features while retaining the general features captured by EfficientNet. Subsequently, we unfroze the EfficientNet layers and performed fine‐tuning with a reduced learning rate, allowing for adjustments to the pre‐trained weights in EfficientNet, thereby enhancing the model's performance on our specific data set.

In both phases, the loss function used was categorical cross entropy, ADAM optimizer (Kingma 2014) was responsible for controlling weight updates, and early stopping was implemented to prevent overfitting. The early stopping criteria were based on validation loss, with a patience parameter defining the number of epochs to wait for improvement before halting the training process (Table 3).

Supporting file S1 *: Model development and training process*.

**Table 3:** Key hyperparameters used in model training.

Hyperparameter	Value/Phase
Learning Rate	Transfer learning: 0.001; Fine‐tuning: 0.0001
Batch size	32
Optimizer	Adam
Dropout rate	0.2
Number of epochs	Transfer learning: 50; Fine‐tuning: 30
Loss function	Categorical cross‐entropy
Dense layer units	128
Early stopping patience	10 epochs
Early stopping monitor	Validation loss

### 2.5 Evaluation

The performance of the model was assessed using precision, recall, and F1 score across multiple classes, as well as overall scores (macro, micro, and weighted). Precision, recall, and F1, for a given class, are given by:

Precision=TP/TP+FP

Recall=TP/TP+FN

F1=2(Precision×Recall)/Precision+Recall

where TP is the number of true‐positive predictions, FP is the number of false‐positive predictions, and FN is the number of false‐negative predictions.

#### 2.5.1 AI Model and Infection Control Auditors' Evaluation for the Quality of HH Performance Comparison

The AI model and two infection control auditors evaluated the HH performance in 22 videos recording separately. The videos used in this comparison were not part of the videos used for model's training and validation.

To interpret the AI model's output and whether an HH procedure was acceptable or not, each video was processed in batches of 16 frames. The model performed inference on each frame within the batch, and a majority voting scheme with a minimum threshold of 12 votes was employed to assign a label to the entire batch. If no consensus was reached, the batch was assigned label 6 (unknown/invalid). This approach mitigated the potential noise in individual frame predictions. By aggregating the number of batches corresponding to each label, we calculate the duration of each hand movement present in the video. Overall performance, whether acceptable or not, was based on performing all steps for a period of at least 1 s for each step.

Two auditors – with experience in dentistry and infection control – used a standard checklist to evaluate the videos recording of dental students performing ABHR. In the checklist, each hand position was identified as accurately done or not. Based on accurately performing all the steps of HH, an overall evaluation of the performance was recorded, and comments were collected.

The agreement referred to the ability of the AI model to provide the same assessment of an event as a human auditor. The level of agreement between the infection control auditors and the AI assessments was calculated using Cohen's kappa.In addition, the sensitivity and specificity of the AI compared to the infection control auditors were calculated.

## 3 Results

A total of 66 videos were recorded. Of these, 55 were allocated as follows: 33 assigned for training the model, 11 for validation, and 11 for testing. For the AI‐infection control auditors comparison experiment, the testing videos were utilized alongside an additional 11 videos, resulting in a total of 22 videos used in the comparison experiment.

### 3.1 AI Evaluation Performance

The evaluation metrics for precision, recall, and F1 score for each class in the test set are presented in Table 4, along with the weighted confusion matrix in Table 5. The results show that the CNN model has learned to differentiate between classes, with an overall F1 score of 0.85. The majority of classes (Classes 0, 2, 3, 5, and 6) have an F1 score between 0.82 and 0.88.

**Table 4:** Evaluation metrics for precision, recall, and F1 score for each class in the test set.

Class	Precision	Recall	F1
0	0.76	0.75	0.76
1	0.94	0.94	0.94
2	0.94	0.61	0.74
3	0.93	0.96	0.94
4	0.89	0.78	0.83
5	0.99	0.89	0.94
6	0.73	0.94	0.82
**Overall (micro)**	**0.85**	**0.85**	**0.85**

**Table 5:** The weighted confusion matrix presenting the classification performance across seven predicted and actual categories.

		Predicted values						
		0	1	2	3	4	5	6
Actual values	**0**	0.91	0.00	0.01	0.00	0.00	0.00	0.08
	**1**	0.01	0.95	0.00	0.00	0.00	0.00	0.03
	**2**	0.07	0.00	0.73	0.00	0.03	0.00	0.16
	**3**	0.00	0.00	0.00	0.89	0.01	0.00	0.10
	**4**	0.02	0.03	0.00	0.00	0.63	0.00	0.32
	**5**	0.05	0.02	0.00	0.00	0.01	0.78	0.13
	**6**	0.02	0.01	0.01	0.03	0.05	0.00	0.88

Class 1 performed best with an F1 score of 0.96. Class 4 significantly underperformed with an F1 score of 0.68.

The weighted confusion matrix presents the classification performance across seven predicted and actual categories. The matrix shows that the model accurately predicted most instances on the diagonal, where true and predicted values match. However, there are notable misclassifications, especially between classes 2 and 6, 0 and 2, and 4 and 6. The overall accuracy of the model is 0.85.

### 3.2 AI – Infection Control Auditors' Comparison and Accuracy

An agreement rate of 90.91% was observed between infection control auditors and the AI model for overall approval of the HH steps. Only two videos where AI identified performance as not acceptable, contrary to auditors' evaluation where those videos were accepted. Cohen's kappa of 0.81 (95% confidence interval [CI], 0.57–1).

The AI algorithm demonstrated sensitivity of 85.7% (95% CI, 57.1%–98.2%) and specificity of 100% (95% CI, 63.1%–100%) in correctly identifying the acceptable HH practice. The model's positive predictive value was 100% (95% CI, 73%–100%), while its negative predictive value was 80% (95% CI, 55.5%–93.5%).

Out of the 22 videos evaluated, infection control auditors approved 14 (64%) of the HH techniques, and AI approved 12 (55%) In one of the videos not approved by the AI, more missed steps were indicated compared to the auditors' evaluation, while in another video, Step 2 occurred for less than 1 s, according to the AI.

Both auditors and AI agreed on the steps missed in the videos, with Step 3 (back of fingers to opposing palm with fingers interlocked) being the most missed step (50%). AI also identified Step 2 (41%) as the second most missed step, while in the auditors' evaluation, all other steps were equally missed (27%). Step 0 (palm to palm) was performed in all the videos.

The checklist sheets filled out by the auditors included comments on the overall evaluation of HH performance quality, such as the organization of the steps, repeated steps, and observations regarding the use of nail polish or accessories.

## 4 Discussion

This study aimed to evaluate the effectiveness of an AI Model in detecting adequate HH performance compared to infection control auditors. The results indicated a strong agreement between the AI algorithm and human evaluators in identifying HH steps and evaluating performance in a dental setting. Notably, the specificity of the system was found to be high. The primary focus was whether the machine learning algorithm could accurately recognize hand movements in HH performance videos. The study demonstrated that the developed AI algorithm is a reliable assessment method for evaluating effective HH practices compared to the gold standard technique of human expert observation.

Compliance with the HH measures plays a crucial role in reducing infection transmission in hospitals and dental clinics (World Health Organization 2009). Yet compliance with HH remains suboptimal (Kato et al. 2021). Studies have shown that HH adherence among dental students was found to be below 50% of the total number of opportunities (Resende et al. 2019). Although dental educators washed their hands 2.31 times more often than students, the HH adherence rate was still less than 70% (Resende et al. 2019).

The quality of performed HH is also highly suboptimum with only 72% of health care workers (HCW) adequately cleaned all hand surfaces after HH training (Szilágyi et al. 2013). This is in accordance with the present study, which found that approximately half of the videos evaluated were considered inadequate and missed important steps, highlighting the need for improved monitoring and feedback mechanisms.

One advantage of infection control auditors observing HH is the availability of detailed feedback (Fitzpatrick et al. 2020; Fujita et al. 2022), as seen in this study where auditors noted aspects such as the sequence of steps, time spent, and the use of accessories or nail polish—details that AI was not trained to detect. However, expert observation can be labor‐intensive, time‐consuming, and subject to bias from the Hawthorn effect, which can introduce up to 65% bias in HH compliance (Geilleit et al. 2018). To reduce this effect, the auditors in this study evaluated recorded videos using a checklist.

AI algorithm development for HH was reported by several studies (Geilleit et al. 2018; Kwok et al. 2015; Lacey et al. 2020). AI systems were cost‐effective methods in improving and monitoring HH. However, limited studies compared the results of the AI algorithm to direct observation by experts (Casaroto et al. 2022), and none was done in the dental setting. Casaroto et al used sensors that obtained infrared images of HH when performed preceding to patients care. The system issued a warning when HH was not performed that helped to remind the HCW about HH. Data were compared to those obtained by human observers, and the electronic system correlated well with the human observer method. Similarly, this study reported that the AI model and infection control auditors agreed in 90% of the cases. Only in 2 videos AI model results did not agree with the auditor's report. In addition, both the AI model and auditors agreed that the most frequently missed step was Step 3. A similar finding was reported by Shi et al. where the rub between fingers was the most omitted (Shi et al. 2023). On the other hand, palm to palm (Step 0) was performed in all the videos in this study and was never missed, similar to previous reports (Kwok et al. 2015).

The dental students who participated in the study were from different clinical years. They received a comprehensive infection control course before starting the clinical training that included a detailed explanation and practice performance of HH techniques. Many studies have noted that dental students have a high level of knowledge in infection control and HH and yet their practice of HH is inadequate (Baier et al. 2020; Meisha 2021). Moreover, students' attitude and practices of HH decreased by increased clinical experience (Yaembut et al. 2016). Competencies decrease over time, and reinforcement courses with practice and feedback are important factors in the retention of clinical skills (Vlasblom et al. 2020). From this perspective comes the value of our research work. It can augment the compliance rate of proper HH among students and HCWs in dental clinics.

Testing the performance of AI in real‐life clinical practice is important. AI is highly dependent on the quality and completeness of the input data. Errors can be introduced during the machine learning process, which may result in misclassification or false outcomes. To avoid this, data annotation was repeatedly reviewed by an expert in infection control. AI results may reflect biases present in the training data, potentially leading to biased outcomes. For example, participants might intentionally avoid occluding their hands when being recorded, which could result in a data set that fails to capture more ordinary HH behaviors.

The F1 scores in certain classes can be attributed to specific patterns of misclassification. 83% of all prediction errors stem from confusion with Class 6, which represents the “unknown/invalid” class. This behavior is expected due to the challenges posed by the boundaries between well‐defined classes and the “unknown/invalid” class. 50% of the models' misclassifications predicted the correct label as their second choice. The effect of these misclassifications is partially mitigated during post‐processing, which considers the model's output confidence in class 6.

Despite being the largest source of misclassifications, the incorporation of an “unknown/invalid” class (Class 6) within our data set enhances the model's adaptability to real‐world applications. In any practical deployment, HH classification models must contend with transitions between movements or activities that may not fit predefined categories, such as pauses between procedures or using hand sanitizer. In the context of dental education, the model must also deal with students who frequently exhibit similar kinds of invalid movements or use inadequate techniques. Without an explicit “unknown/invalid” class, the model would struggle with generalizing to unseen movements. Samyak et al. address this by setting thresholds for confidence scores, below which a movement is deemed invalid (Shrimali and Teuscher 2023). The problem with confidence thresholds is that deep neural networks are known to be overconfident, and even after calibrating, they can perform poorly with samples outside the training set (Jang 2024). Other approaches involve smoothing softmax probabilities to manage uncertainty (Wang et al. 2022). However, these methods fail to exploit the discriminative representations for unknowns that the model may have learned (Jang 2024). By integrating an “unknown/invalid” class, we streamline the model's decision‐making process, enabling it to reject ambiguous inputs and ultimately improving the robustness of predictions in practical scenarios. Despite these challenges, the F1 score achieved in this study was comparable with previous attempts using CNNs across various datasets (Xie et al. 2022; Nagar et al. 2022; Prakasa 2022).

Future improvements could involve using more complex models and diverse settings to enhance generalizability. For instance, temporal relations between frames can be leveraged through 3D CNNs (Wang et al. 2022) and CNN + LSTM (Cikel 2021) architectures. However, greater model complexity increases computational costs, training time, and data requirements, and has a higher risk of overfitting (Elsts 2022), making real‐world deployment challenging due to memory and performance constraints. This paper demonstrates that the available modeling techniques are already impactful in the field of HH.

The use of AI in monitoring HH can be expensive and may incur initial expenses related to equipment, installation, and maintenance. However, in the long run, investing in AI technology could prove cost‐effective when considering the time saved and the expenses associated with employing human experts. Nevertheless, concerns regarding data confidentiality, ownership, and privacy are critical factors influencing the acceptance of intelligent intervention (Meng et al. 2022).

Several limitations are inherent in our study. First, errors in AI model may have occurred due to mislabeling events, although efforts were made to mitigate this risk by thoroughly reviewing the steps and annotations multiple times. Additionally, the study was conducted at a single location, which may limit the generalizability of the findings to other clinics without further detailed training of the AI model. Expert observation in the study involved watching recorded videos, allowing auditors the opportunity to rewatch and review the footage as needed. This method contrasts with real‐time observation of HH practices, which typically involves immediate feedback and correction, highlighting a potential divergence from real‐world practice.

Future research could focus on validating the AI model in various clinical settings to evaluate its generalizability and effectiveness across different environments. It should also investigate the feasibility of using an AI model for long‐term monitoring of HH and assess sustainability and continuous improvement in compliance rates, infection control outcomes, and patient safety measures over time. Additionally, it could explore the integration of the AI model with real‐time feedback mechanisms to provide immediate guidance to HCWs during HH procedures, potentially enhancing adherence and performance.

## 5 Conclusion

AI models can assess the accuracy of HH in a manner comparable to infection control auditors. The AI algorithm tested in this study demonstrated high sensitivity and specificity, supporting its use as a reliable tool for mentoring and evaluating HH practices in dental clinics. These findings highlight the potential of AI to complement traditional methods and enhance HH performance, a critical aspect of infection control for safe dental practice. Further research and testing of various AI models in real‐world clinical settings are necessary to fully maximize their effectiveness in promoting patient safety and minimizing infection risks.

## Author Contributions

**Salwa A. Aldahlawi:** conceptualization, analysis and interpretation of data, statistical analysis, drafting the manuscript**. Amr H. Almoallim:** AI algorithms designing, acquisition of the data, critical revision for important intellectual content**. Ibtesam K. Afifi:** study supervision, analysis and interpretation of data, critical revision for important intellectual content. All authors critically revised the manuscript and approved the final revision.

## Ethics Statement

Ethical approval was obtained from the Institutional Review Board of Umm Al‐Qura University (No. HAPO‐02‐K‐012‐2024‐02‐2046).

## Conflicts of Interest

The authors declare no conflicts of interest.

## Supporting information

Supporting file S1.

## Data Availability

The data that support the findings of this study are openly available in zenodo at 10.5281/zenodo.13937448.
