# Supplementary material for: Artificial Intelligence and Hand Hygiene Accuracy: A New Era in Infection Control for Dental Practices
Source: Clin Exp Dent Res. 2025 May 26;11(3):e70150. doi: 10.1002/cre2.70150 (PMC12104871; doi:10.1002/cre2.70150)
Supplement: Supplementary file 1 — Supporting file S1. [file CRE2-11-e70150-s001.docx]

***Supplementary file S1: Model development and training process***

The AI model employed a **Convolutional Neural Network (CNN),** a type of deep learning architecture well-suited for analyzing visual data. To enhance its decision-making capabilities, the model included a custom classifier composed of five fully connected (dense) layers. These layers progressively interpret and classify visual patterns extracted from input data. The use of the **ReLU (Rectified Linear Unit**) activation function enables the model to efficiently focus on relevant features while minimizing the influence of less important information. This architecture allows the AI system to accurately identify and interpret complex visual behaviors, making it a valuable tool for applications such as automated infection control auditing.

The model training was conducted in two phases to maximize efficiency and performance. In the initial phase, transfer learning was applied by freezing the pre-trained EfficientNet layers and training only the newly added dense layers. This allowed the model to retain general visual features learned from large datasets while acquiring task-specific knowledge. In the second phase, all layers—including those of EfficientNet—were unfrozen, and fine-tuning was performed with a reduced learning rate to make small, precise adjustments to the entire model. This refinement improved the model's performance on the specific dataset.

During both phases, **categorical cross-entropy** was used as the loss function. This function measures how far the model's predictions are from the actual labels, and guides the training process by penalizing incorrect predictions more heavily. A lower loss value indicates better model performance. The **ADAM optimizer** was used to update the model's internal parameters in response to the calculated loss, helping the model learn efficiently.

To avoid **overfitting**—a situation where the model performs very well on training data but poorly on new, unseen data—**early stopping** was employed. This technique monitors the model's performance on validation data and automatically halts training when no further improvement is detected after a set number of iterations (defined by a "patience" parameter). This ensures the model remains generalizable and performs well on real-world inputs.

This two-phase training process makes the model both **efficient** and **effective**. It leverages existing powerful models to save time and resources, while still tailoring the model to the specific needs of the project. The use of best practices like early stopping and careful fine-tuning ensures that the model doesn’t just memorize data but learns in a way that generalizes well to real-world scenarios.
